# From Seed to Young Plant: A Study on Germination and Morphological Characteristics of *Crateva tapia* L. (Capparaceae)

**DOI:** 10.3390/biology14121729

**Published:** 2025-12-02

**Authors:** Rosemere dos Santos Silva, Flávio Ricardo da Silva Cruz, Maria Lúcia Maurício da Silva, Maria das Graças Rodrigues do Nascimento, Edlânia Maria de Sousa, Joel Maciel Pereira Cordeiro, João Henrique Constantino Sales Silva, Edna Ursulino Alves

**Affiliations:** 1 Department of Biosciences, Center for Agrarian Sciences, Federal University of Paraíba, University Campus II, Areia 58397-000, PB, Brazil; 2 Department of Soils and Rural Engineering, Center for Agrarian Sciences, Federal University of Paraíba, University Campus II, Areia 58397-000, PB, Brazil; flavioricardocruz@gmail.com; 3 Department of Agricultural and Exact Sciences, Center for Human and Agricultural Sciences, State University of Paraíba, Catolé do Rocha 58884-000, PB, Brazil; eumaria.agronomia@gmail.com; 4 National Institute of the Semiarid (INSA), Campina Grande 58434-700, PB, Brazil; graca.agronomia@gmail.com; 5 Department of Agriculture, Federal University of Lavras, Lavras 37200-900, MG, Brazil; edlania.maria@hotmail.com; 6 Center for Exact and Natural Sciences, Federal University of Paraíba, University Campus I, João Pessoa 58051-900, PB, Brazil; joelmpcordeiro@gmail.com; 7 Postgraduate Program in Agronomy, Federal University of Paraíba, University Campus II, Areia 58397-000, PB, Brazil; ursulinoalves@hotmail.com; 8 Department of Plant Science and Environmental Sciences, Center for Agrarian Sciences, Federal University of Paraíba, University Campus II, Areia 58397-000, PB, Brazil

**Keywords:** native Caatinga species, germination, fruit and seed morphometry, water absorption, vigor

## Abstract

*Crateva tapia* L. is a native tree species of the Caatinga biome, with medicinal and allelopathic properties. This study aimed to characterize the biometric and morphological traits of fruits, seeds, seedlings, and young plants of *C. tapia*, as well as to evaluate the germination pattern of its seeds under different temperatures, in order to understand the reproductive strategies and optimal conditions for its propagation. The results are intended to support conservation efforts, ecological restoration, and the sustainable use of the species within the Caatinga biome. The research was carried out at the Seed Analysis Laboratory of the Federal University of Paraíba, Campus II, Areia–PB, Brazil. The biometric data were obtained from 100 fruits and 100 seeds obtained from eight mother plants. The imbibition curve was determined from the weight of the seeds during 216 h at different temperatures, and the germination test was performed concomitantly. The fruits were morphologically described regarding the external and internal aspects of the pericarp. The seeds were described according to their consistency, color, texture, shape, hilum, and embryo. The fruits of *C. tapia* vary in size, fresh mass, and number of seeds, characterized as amphisarcidium, indehiscent, and polyspermic. The seeds are small, brown, reniform, and biting, with a cotyledonary embryo with a poorly differentiated hypocotyl–radicle axis, with a well-developed hilum and surrounded by a fleshy mesocarp. The germination of *C. tapia* seeds is epigeal-phanerocotyledonous; the seedlings have white axial roots containing absorbent hairs.

## Simple Summary

*Crateva tapia* L., commonly known as trapiá, is a tree species native to the Caatinga biome, with medicinal, ornamental, and forage potential, and is recommended for the recovery of degraded areas. This study aimed to characterize the biometric and morphological traits of fruits, seeds, seedlings, and young plants of *C. tapia* (Capparaceae), as well as to evaluate the seed germination pattern under different temperatures, in order to understand its reproductive strategies and optimal propagation conditions. A total of 100 fruits collected from different trees and 100 seeds randomly selected from these fruits were analyzed and described in terms of external and internal characteristics. To assess germination, seeds were maintained in chambers under controlled temperature and light conditions and monitored daily for 24 days. The fruits showed wide variation in size, mass, and number of seeds, being amphisarca, with a succulent pericarp, yellow externally and white internally. The seeds are exalbuminous, reniform, brown, and bitegmic, containing a cotyledonary embryo with a poorly differentiated hypocotyl–radicle axis. The imbibition process followed a triphasic pattern within the temperature range of 20 to 35 °C, with the highest germinative efficiency observed at 30 °C. The results contribute to conservation efforts, ecological restoration, and the sustainable use of this species within the Caatinga biome.

## 1. Introduction

*Crateva tapia* L., known as trapiá, is a species of the Capparaceae family (the same as the capers, *Capparis spinosa* L.), native to the Cerrado and Caatinga biome in Brazil, commonly found in dry forests, dune forests, kermes ore, riparian forests, carnaubal, mangrove swamps, and coastal forests [1]. It is an arboreal species, being indicated for afforestation and restoration of degraded areas [2]. Its bark and leaves have antioxidant properties against free radicals and can be used in the food and pharmaceutical industry. The leaf extract has intense allelopathic activity and can be explored as a natural herbicide [3].

The plant annually produces a large amount of obovoid, succulent, indehiscent, green (immature), and yellow (ripe) fruits, which, together with the leaves and bark, have medicinal uses with anti-inflammatory, analgesic, and antitumor properties [4].

Studies on biometrics and morphology of fruits and seeds, as well as the seedlings’ development, can help in the management and seedling production that are indicated for environmental restoration, genetic conservation, and species identification [5,6,7]. This knowledge provides essential information to identify intraspecific and interspecific variations related to biotic, abiotic, and genetic factors and genotype–environment interaction [8,9]. It also contributes to the identification of seeds and young plants related to the successional development of a species [10,11].

Biometric attributes such as the size and mass of fruits and seeds are intrinsic factors linked to the reproductive strategies of plants, which interfere in the establishment, survival, and growth of pioneer species [12]. In addition, ranking by size or mass can be applied to standardize the emergence of seedlings and acquisition of seedlings with standardized sizes and greater vigor [13]. Morphometric studies in species of the Capparaceae family are incipient, and only one biometric article with *Capparis flexuoxa* L. is found in the literature [14].

The characterization of the seed imbibition stages, particularly at different temperatures, is important to demonstrate the germinative behavior when making decisions about osmotic conditioning, given that the three-stage pattern and the water imbibition speed are different between species [15,16].

Seed germination opens the way for a species to be established in the field, which starts with water imbibition by the seed tegument, triggering physiological and metabolic changes [17,18]. Nevertheless, the temperature is a limiting factor in this process because it interferes in biochemical reactions and the water absorption speed [19]. Tropical native species can germinate over a wide temperature range depending on the biome and region, but most species germinate satisfactorily between 20 and 35 °C [20]. Low temperatures promote slow respiratory rates, while high temperatures can lead to embryo death [21].

This study aimed to characterize the fruits and seeds of *C. tapia* biometrically, describe the morphology of fruits, seeds, seedlings, and young plants, and characterize the germination pattern of its seeds at different temperatures. This is the first study of the species that integrates biometric and morphological analyses, covering everything from fruits and seeds to seedlings and young plants, associated with the evaluation of seed germination behavior. This multidimensional approach allows for a better understanding of the initial development and ecological adaptation of the species, providing unprecedented information that can support conservation and propagation programs, as well as assist in taxonomic and evolutionary studies, potentially indicating adaptive processes or even cryptic species within the group.

## 2. Materials and Methods

The fruits of *Crateva tapia* were manually collected from the canopy of eight mother trees, maintaining a minimum distance of 50 m between them to reduce possible kinship relationships. Only adult, healthy, and vigorous specimens were selected, located in the municipalities of Remígio and Esperança, state of Paraíba, Brazil, in 2017. Immediately after fruit collection, the experiment was carried out at the Seed Analysis Laboratory of the Departamento de Fitotecnia e Ciências Ambientais of the Centro de Ciências Agrárias of the Universidade Federal da Paraíba. The seeds were manually extracted from the fruits, placed in plastic buckets to ferment for 3 days in the pulp itself, then washed in running water to remove the aril as recommended by [22], and dried on paper towels under a shaded environment for 3 days. Biometric analyses started as soon as the fruits arrived at the LAS and after drying of seeds. The following traits were evaluated:

### 2.1. Water Content

Determined by the oven-drying method at 105 ± 3 °C for 24 h [23], with four samples of 10 seeds, and results expressed in percentage.

### 2.2. Fruit and Seed Biometrics

A random sample of 100 intact and ripe fruits were collected, and the length (longitudinal axis), width (equatorial line), fresh mass, and the number of seeds per fruit, width, thickness, and mass of 100 seeds measured. Measurements were made with a digital caliper of 0.01 mm precision (Jomarca, Guarulhos, SP, Brazil) and an analytical scale of 0.0001 g precision (Bioprecisa-JA3003N, Bioprecisa, Curitiba, PR, Brazil).

### 2.3. Fruit Morphology

The fruits of *C. tapia* were morphologically characterized based on external and internal features, including pericarp, texture, consistency, color, sheen, shape, dimensions, number of seeds, and type of dehiscence.

### 2.4. Seed Morphology

One hundred seeds were randomly collected to describe the external and internal morphology. For that, seeds were immersed in distilled water for 72 h to facilitate the removal of the seed tegument. The intact and sectioned seeds were evaluated with a binocular magnifying glass, and the photographs were taken with a stereomicroscope (ZEISS Stereo Discovery V20 Model, Zeiss, Oberkochen, Germany). A longitudinal cut was made in the embryo with a scalpel blade to observe the internal structures of the seeds for better visualization of the embryo. The external traits observed were color, texture, integument consistency, seed size, and shape. The internal characteristics of the embryo were also observed (cotyledons, hypocotyl–radicle axis, plumule, central cylinder, and the number of integuments or endosperm). The nomenclature used was based on the literature of Glória [24], Vidal & Vidal [25], and Barroso et al. [26].

### 2.5. Water Absorption Curve

Four replicates with 25 seeds were selected for each temperature treatment, weighed on a digital analytical scale (0.0001 g precision), distributed on paper towel rolls (Germitest), and placed in a Biological Oxygen Demand (BOD, Eletrolab, São Paulo, SP, Brazil) germination chamber with a photoperiod of 8/16 h of light and dark, respectively, provided by four 20 W daylight type lamps, and regulated at constant temperatures of 20, 25, 30, 35, and 40 °C for 4 days. Seeds were weighed every 2 h for 12 h and, after this period, every 12 h until the primary root protrusion of 1% of the seeds, following the methodology proposed by Albuquerque et al. [27]. The water content for each evaluation day was determined based on the initial weight of the seeds, according to the formula described by Barros Neto et al. [28].

P2 = (100 − A)/(100 − B) × P1, where A = initial seed water content (wet basis); B = wanted water content; P1 = initial seed weight (g); P2 = final seed weight (g).

### 2.6. Germination Curve and Normal Seedling Development

The germination evaluations started on the fourth day of imbibition when the primary root protrusion of at least one seed was observed, with observations taken every 24 h. The variables evaluated were the final germination percentage, the first germination count, the germination speed index (GSI) [29], root and shoot length (cm seedling^–1^), root and shoot dry weight (g seedling^−1^), percentage of hard seeds, and abnormal seedlings. Counting of normal seedlings occurred on the ninth day of the test and ended on the 24th day, with at least 50% of the seedlings formed.

### 2.7. Morphological Description of Germination, Seedling, and Young Plant

Descriptions of the structures during germination of 100 seeds and seedling formation were performed at each stage of development. Records were taken daily of the seeds, swelling until the formation of the first pairs of cotyledonary leaves. Records of the young plant started from the hypocotyl emergence until the senescence of cotyledons. The temperature of 30 °C was used for the germination morphology.

For the descriptions of young plants, 100 seeds were sown at 2 cm depth in polypropylene tubes of 175 mL containing Basaplant substrate (100%) (Base Substratos Ltda, Artur Nogueira, SP, Brazil). The tubes were distributed in a protected environment and watered daily for 76 days, when three pairs of eophyll and cotyledon started to senesce.

The type of emergence and root (color, presence of secondary and tertiary roots), collar, hypocotyl (color and shape), cotyledons (shape, color, and texture), epicotyl (color and shape), eophylls (of 1st and 2nd order), and stem were characterized. The terminology used to describe the external characteristics of seedlings and young plants was based on the classification of Glória [24], Vidal & Vidal [25], Barroso et al. [26], and Gonçalves & Lorenzi [30]. As mentioned earlier, all morphological characteristics described and illustrated were performed with a binocular magnifying glass (Physis, Santo André, SP, Brazil), a digital camera (Sony^®^ SEL 1855, Tokyo, Japan), and a stereomicroscope (Zeiss, Oberkochen, Germany).

### 2.8. Statistical Analysis

Biometric data of fruits and seeds were submitted to descriptive statistical analysis and the mean, standard deviation, coefficient of variation, maximum and minimum values, asymmetry, kurtosis, and relative frequency distribution calculated. Shapiro–Wilk test was also performed. For the germination data, analysis of variance and the Tukey test (*p* < 5%) were used to compare the means. The statistical software Sisvar 5.6 was used [31].

## 3. Results

### 3.1. Biometrics of Fruits and Seeds

**Figure 1:**
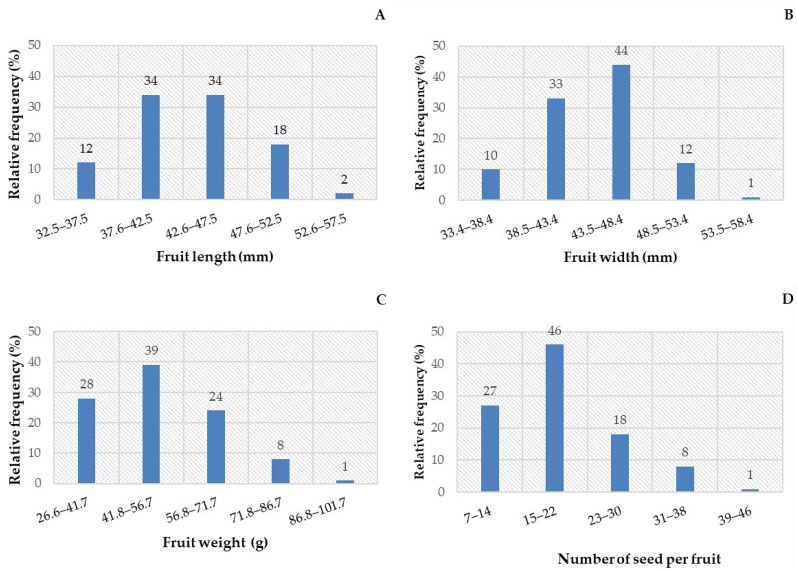
Relative frequency distribution of fruit length (**A**), fruit width (**B**), fruit weight (**C**) and the number of seeds per fruit (**D**) in *Crateva tapia*.

Wide distribution was verified between the frequency classes of *C. tapia* fruits (Figure 1), in which fruit length ranged from 32.5 to 57.5 mm (Figure 1A), width from 33.4 to 58.4 mm (Figure 1B), and fresh mass from 26.6 to 101.7 g (Figure 1C), whereas the number of seeds per fruit ranged from 7 to 46 (Figure 1D).

The length and width values varied (Figure 1A,B). However, two classes represented 68% of the fruits with a length of 37.6 to 47.5 mm, while 44% measured 43.5–48.4 mm in width. For the fruit mass, the variation was of 91% frequency classes, with values between 26.6 to 71.7 g (Figure 1C), and the number of seeds per fruit varied in 46% of the sample, from 15 to 22 seeds (Figure 1D).

The mean length of *C. tapia* fruits was 4.33 cm, with a maximum value of 5.33 cm and a minimum of 3.25 cm (Table 1), whereas for width, a mean value of 4.37 cm, with a maximum of 5.42 cm and a minimum of 7.71 cm, was obtained. The mean fresh fruit mass recorded was 51.47 g, reaching a maximum value of 97.59 g and a minimum of 26.69 g, with a mean of 19.12 seeds.

Standard deviation values ranged between 0.43 and 14.64, with the length, width, and the number of seeds per fruit having the lowest dispersion. For the curve symmetry, data closer to zero were verified for length and width but still asymmetric to the left. The mass and number of seeds per fruit had positive asymmetry, given the right-skewed distribution.

The kurtosis coefficient had values below and above 3, which indicates that the distribution was platykurtic for length and width, leptokurtic for mass, and mesokurtic for seed number. According to the Shapiro–Wilk test, fruit length and width had a normal distribution. The coefficient of variation indicated the length and width of fruits as variables with excellent experimental precision (<20%).

**Figure 2:**
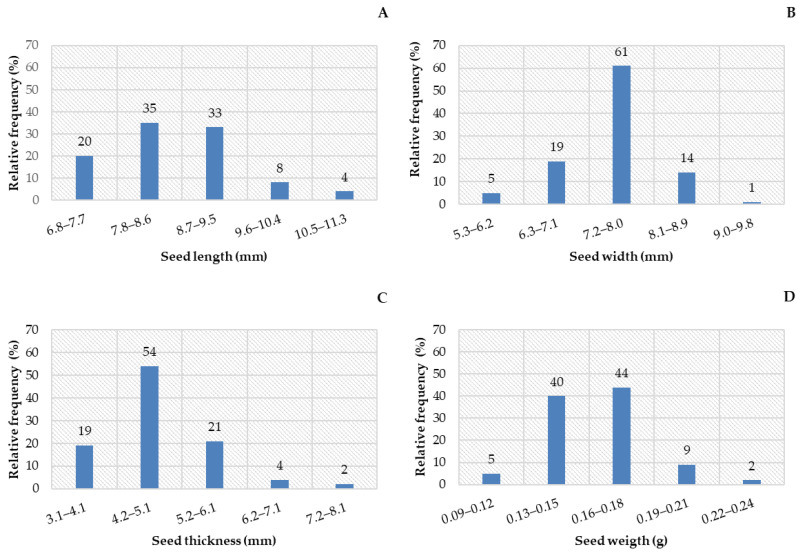
Seed length (**A**), seed width (**B**), seed thickness (**C**), and mass of 100 seeds (**D**) of *Crateva tapia*.

The relative frequency distribution of seed length, width, thickness, and mass was better expressed in eight classes (Figure 2). When these biometric data were observed, it was found that the mean length of *C. tapia* seeds varied between classes, with the highest percentage of 68% from 7.8 to 9.5 mm (Figure 2A); 61% of the seeds ranged from 7.2 to 8.0 mm width (Figure 2B), and thickness was mainly distributed in the second class (4.2 and 5.1 mm), representing 54% of the sample (Figure 2C). For the fresh mass of seeds, 84% weighed from 0.13 to 0.18 g (Figure 2D).

Seeds of *C. tapia* had a mean length of 0.85 cm and ranged from 0. 68 to 1.08 cm (Table 2); the mean width was 0.74 cm, with a maximum of 0.90 cm and a minimum of 0.53 cm. The mean thickness was 0.49 cm, with a maximum of 0.78 cm and a minimum of 0.32 cm, while the mass of 100 seeds was 0.15 g, with values that ranged from 0.09 to 0.23 g. The standard deviation confirms the data homogeneity, which ranged from 0.02 to 0.69. The coefficient of variation (CV) for width was considered low, and for the other variables, it was considered medium.

According to the symmetrical distribution of the curve, the length, thickness, and fresh mass of seeds had positive asymmetry and negative width. As observed for the curve flattening degree (kurtosis), the length had a distribution function more flattened than normal, therefore characterized as platykurtic. In contrast, the other traits were leptokurtic, as they had a fatter tail. Based on the Shapiro–Wilk test, the length and fresh mass of *C. tapia* seeds had a normal distribution.

### 3.2. Morphological Aspects of the Fruit and Seed

**Figure 3:**
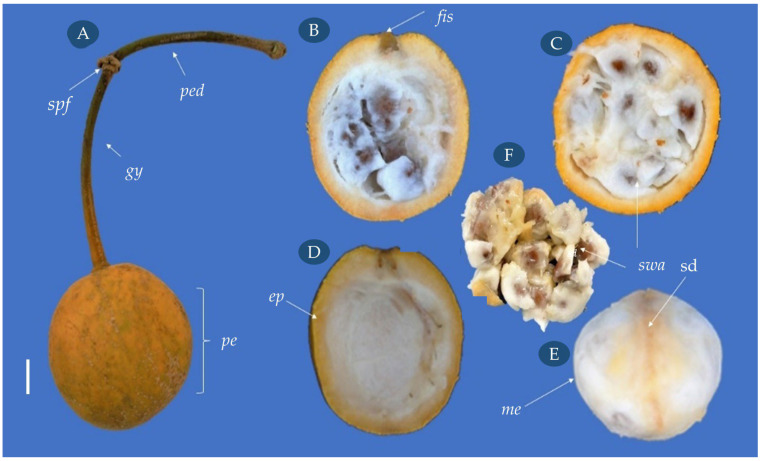
External and internal aspects of *Crateva tapia* fruits. Fruit (**A**), longitudinal section (**B**), cross-section (**C**), inner view of the epicarp (**D**), mesocarp (**E**), seeds surrounded by the aril (**F**). *ped*–peduncle, *spf*–scar of the floral pieces, *gy*–gynophore, *pe*–pericarp, *ep*–epicarp, *fis*–floral insertion scar, *me*–mesocarp, *swa*–seed with aryl, *sd*–dorsal suture. Scale bar = 1 cm.

Fruits of *C. tapia* had 4.33 cm length, 4.37 cm width, 51.47 g of fresh mass, and approximately 19 seeds, characterized as the amphissarcidium type, having globose, obovoid, or oblong shape, indehiscent, polysmermic, and without replum (Figure 3).

The fruit has a peduncle and long gynophore of woody aspect (Figure 3A). The gynophore consists of a structure in which the portion of the receptacle bearing the ovary is elongated. There is a scar between the peduncle and the gynophore, where the floral parts were inserted, and an insertion scar from fruit detachment of the gynophore can be observed (Figure 3B).

The fleshy and crunchy pericarp contains a central cavity where the seeds have adhered to the mesocarp, and is succulent and white with a pungent odor and sweet taste (Figure 3B–D). The epicarp is thick, consistent, fleshy, externally yellow, and internally white (Figure 3D). A line surrounds the mesocarp, consisting of the dorsal suture of placental origin (Figure 3E). In the immature stage, the epidermis is dark green, turning yellow and orange-yellow in more advanced maturation processes. The fruits can be found in the form of racemes and, when ripe, they detach from the long woody peduncle.

**Figure 4:**
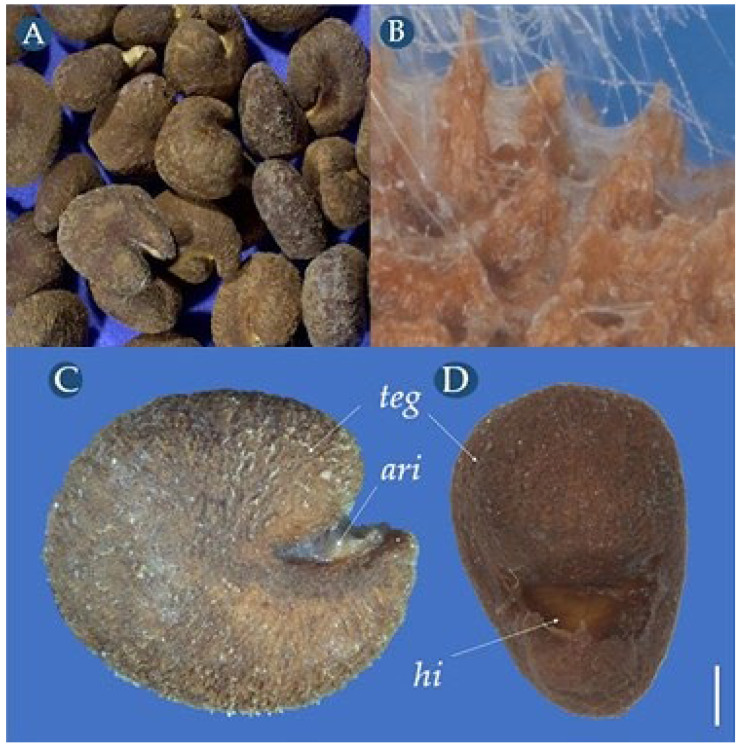
External aspect of *Crateva tapia* seeds. Seeds (**A**), view of cuneiform tubercles (**B**), side view (**C**), ventral view (**D**). Scale bar = 0.15 cm.

Seeds of *C. tapia* are brown and can range from yellow (5Y 8/12), orange (2.5YR 6/14), and red (2.5YR 4/8) to brown (5YR 4/4) and are small, with a reniform shape (Figure 4A), measuring 0.83 cm in length, 0.74 cm in width, and 0.49 cm in thickness, and weighing 0.15 g, being, therefore, lateral-compressed. The integumentary surface is woody, consisting of tiny and irregular coniform tubercles on the back and faces (Figure 4B). The hilum is large, located in the ventral region, between the radicle and the cotyledons, where a clear groove can be seen, which ends in a deeper cavity in the center, containing traces of the aryl (Figure 4C,D). The seeds are completely surrounded by aryl, and the micropyle is inconspicuous.

**Figure 5:**
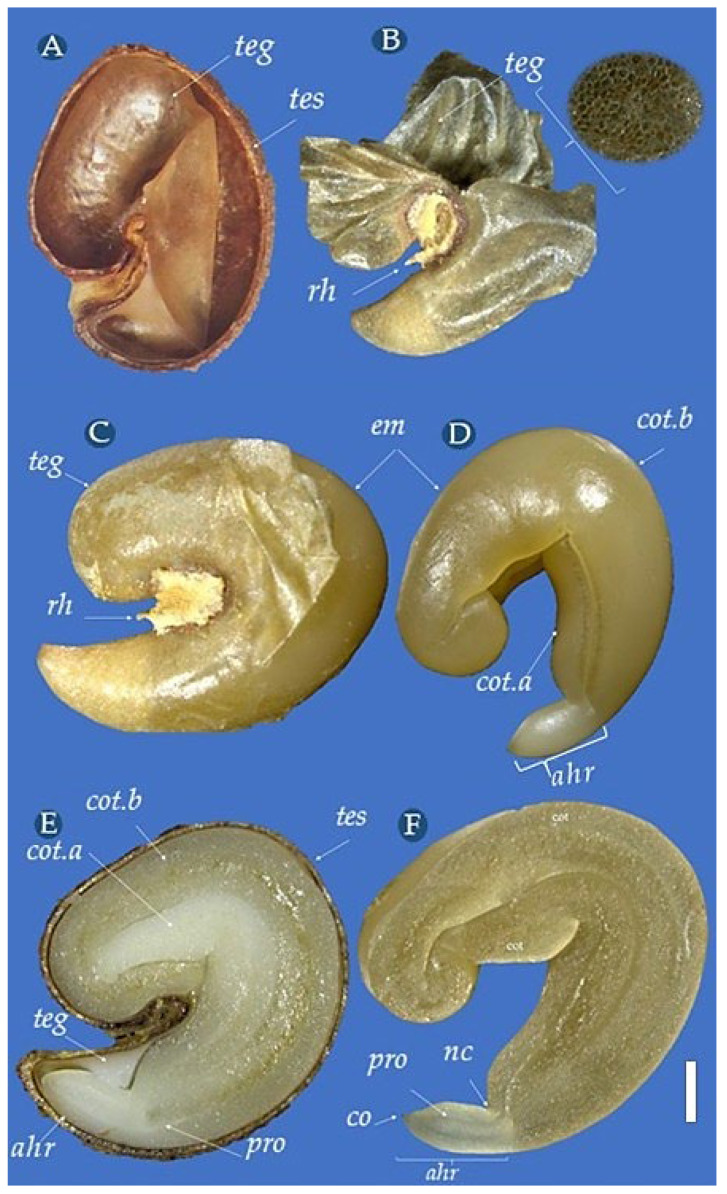
Internal aspects of *Crateva tapia* seeds. Tegument internal view (**A**), tegmen aspect (**B**), view of embryo with and without tegmen (**C**,**D**), longitudinal section of the seed and embryo (**E**,**F**). *ahr*–hypocotyl–radicle axis, *em*–embryo, *pro*-procambium, *te*–testa, *teg*–tegmen, *rh*–hilar region, *cot.a*–smaller cotyledon, *cot.b*–larger cotyledon, *nc*–cotyledonary node, *co*–coif. Scale bar = 0.15 cm.

The seeds are bitegumented with a thin and firm testa (Figure 5A). The tegmen is thin and membranous, with a milky or brownish color and a velvety texture (Figure 5B). Between the apex of the radicle and the ventral cotyledon, an intercellular space is probably filled with air, which gives the integument a milky color. In non-hydrated seed, the tegmen remains attached to the embryo, and after imbibition, it expands to the interior of the testa, releasing space for the radicle elongation.

The cotyledonary embryo is semi-transparent, white or cream-colored, has two folded cotyledons, parallel, smooth, thick, extended, and truncated at the ends, and due to the seed shape, it has different sizes, with the dorsal part larger (Figure 5C,D). The hypocotyl–radicle axis is poorly developed, slightly curved, and delimited by the cotyledonary node, having a reduced size corresponding to approximately 1/3 or less of the seed length, and is located in the basal region or smaller end of the seed (Figure 5E,F). In Figure 5F, a layer of meristematic tissue (procambium) can be seen from the paradermal longitudinal section in the central and bilateral region that follows the entire embryo, which acts in the formation of the xylem and phloem, responsible for the transport of crude and elaborated sap in vascular plants.

### 3.3. Water Absorption Curve and Seed Germination

**Figure 6:**
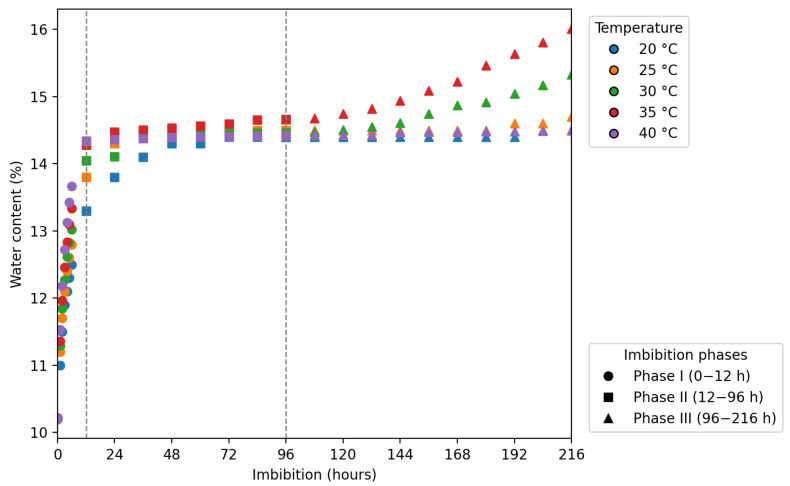
Water imbibition curve of *Crateva tapia* seeds at different temperatures.

The initial water content of *C*. *tapia* seeds after processing and a three-day natural drying period was 10.2%. The water absorption curve of *C. tapia* showed three stages for seeds incubated at temperatures from 20 to 35 °C (Figure 6). In the first 12 h, there was a rapid increase in the water content of seeds from all treatments, characterizing stage I, marked by rapid water absorption. From 12 h onwards, stage II in seeds conditioned at temperatures of 20, 25, 30, and 35 °C starts, in which the water absorption decreases continuously until germination. However, in seeds submitted to the temperature of 40 °C, it was impossible to differentiate the second and third stages. The third stage of germination started at 96 h of imbibition, marked by the resumption of embryonic axis growth, with emphasis on the protrusion of the primary root of at least one seed at temperatures of 20, 25, 30, and 35° C, and germination remained low (<8) during the 216 h.

**Figure 7:**
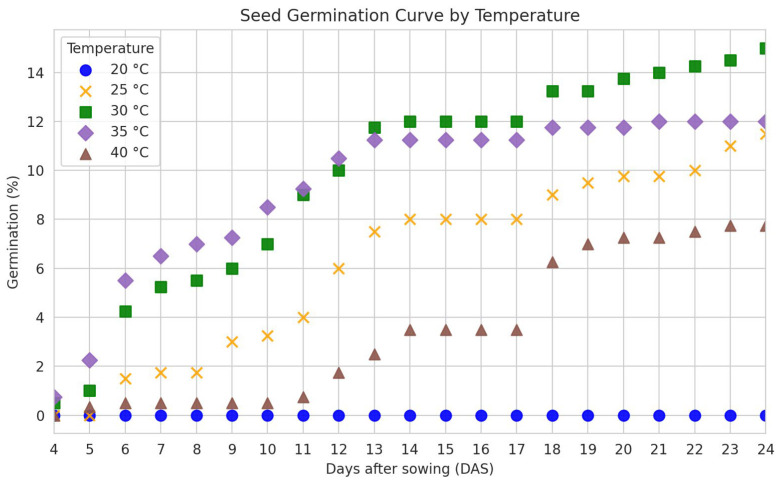
Germination of *Crateva tapia* seeds at different temperatures.

As observed in Figure 7, there was no development of normal seedlings at 20 °C during this period, and the temperatures of 30 and 35 °C were more favorable for seed germination. At 40 °C, germination was low and late, with normal seedlings formed from the 15th day onward.

According to the analysis of variance summary (Table 3), there was a significant effect for percentage, first count, germination speed index, length, and dry mass of roots and shoots of *C. tapia* seedlings up to 1% probability by the F test.

The highest germination (50%), germination speed index (1.15), root and shoot length (0.013 and 0.031 cm, respectively), root and shoot dry mass (0.004 and 0.010 g, respectively) were obtained at 30 °C, whereas for the first count, the highest values were obtained at 35 °C (Table 4). The percentage of hard seeds and abnormal seedlings did not differ statistically; at 20 °C, 100% of hard seeds were obtained, whereas at 40 °C, it was observed that 69% of seeds were hard and 31% of seedlings were abnormal (Table 4).

### 3.4. Germination, Normal, Abnormal Seedling, and Young Plant Morphology

**Figure 8:**
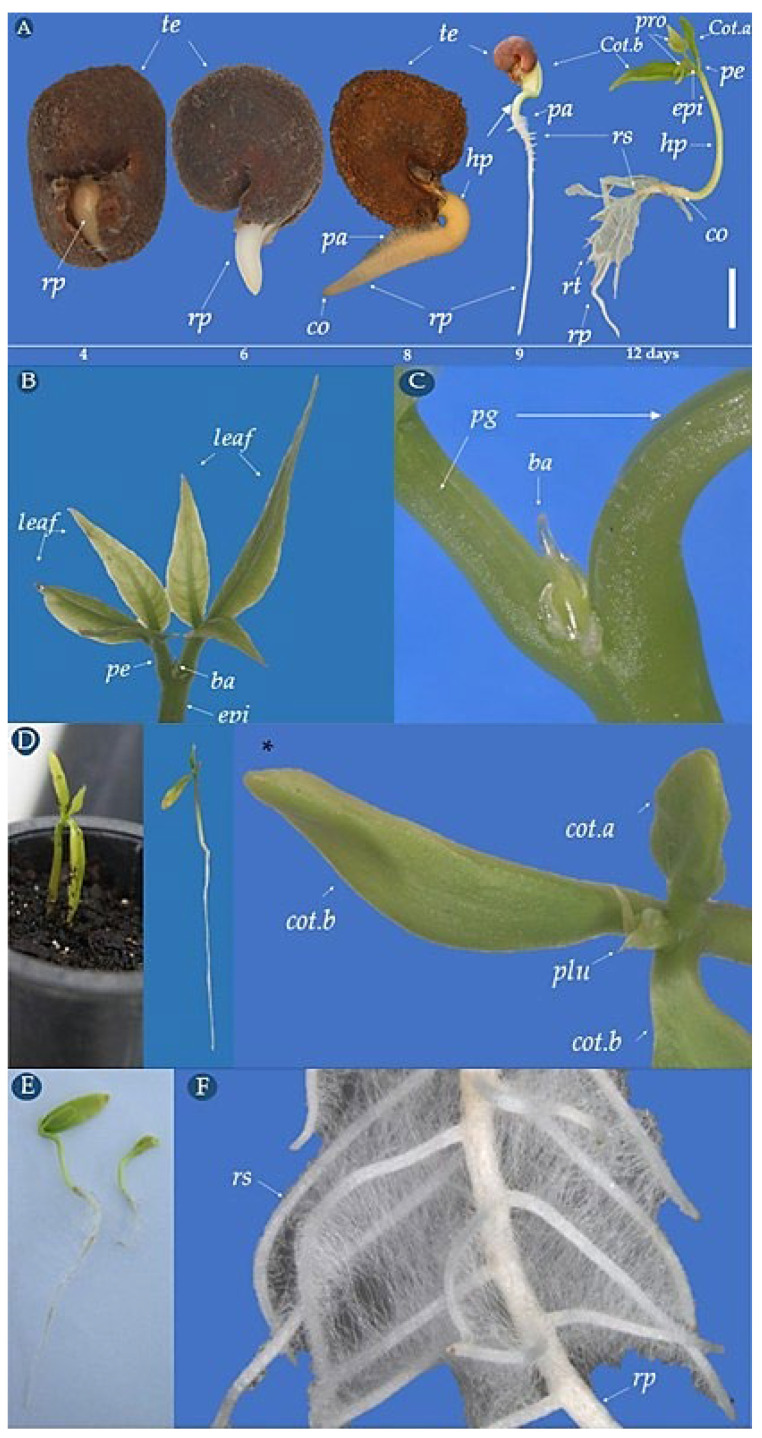
Aspects of germination and normal seedlings of *Crateva tapia* from 4 to 12 days after sowing (**A**), amplified detail of the protophill (**B**), apical bud (**C**), cotyledons and trichotyledonary seedling * (**D**), seedlings of the same seed (**E**), roots (**F**). *rc*–root cap, *cot*–cotyledons, *epi*–epicotyl, *leaf*–leaflets, *ba*–apical bud, *hp*–hypocotyl, *cr*–capillary roots, *pe*–petiole, *pro*–protophill, *te*–tegument, *rp*–primary root, *rs*–secondary root, *rt*–tertiary root, co–collar (hypocotyl–root junction), *spe*–sulcate petiole. * Tricotyledonary seedling. Scale bar = 2.2 cm.

The *C. tapia* seeds showed epigeal-phanerocotyledonary germination, starting after 96 h of imbibition and characterized by the primary root emission, which breaks in the hilar region, initially having a white color, short, wide, and hairless shape, with a white or hyaline root cap (Figure 8A). From the sixth to the seventh day after sowing, an extension of the epidermal cells called absorbent hairs or piliferous zone could be observed. The curved green hypocotyl was cylindrical, hairless, and thick. From the ninth day, it was possible to observe the presence of secondary roots. After 10 days, the hypocotyl became more vertical to the longitudinal axis, raising the cotyledons above the substrate, allowing the complete loosening of the integument. The cervix became noticeable due to the thickening between the primary root and the hypocotyl, which acquired a whitish color.

The cotyledons are fleshy, green, with sulcate petioles, with a larger one folded in the dorsal seed region and a smaller one in the ventral region (Figure 8D). In two plants, the presence of three cotyledons was observed, probably due to mutation that may be related to their ancestral state (Figure 8D).

After 11 days, the epicotyl was 1.0 cm in length, and the hypocotyl was 5.5 cm in length. The integument was detached from the cotyledons. The primary root was 6.5 cm in length, with secondary and tertiary roots denoting an axial root. After 13 days, the epicotyl was 2.1 cm in length, the primary root was 8.5 cm in length, and the cotyledons became perpendicular, showing the protophills and the apical bud with hyaline ends (Figure 8C). A seed was observed to have two embryos, one of which gave rise to one normal seedling, with the other containing all its essential structures, although with reduced cotyledons (Figure 8E).

**Figure 9:**
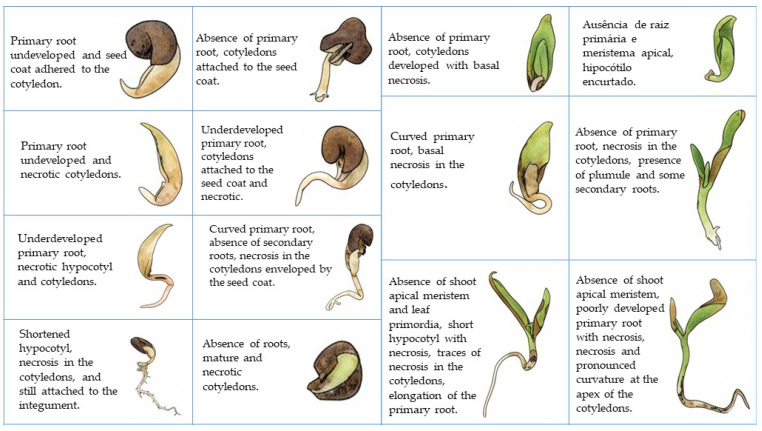
Aspects of abnormal seedlings of *Crateva tapia,* 12 days after sowing.

Many abnormalities were found in the seedlings 12 days after sowing, such as tegument adhered to the cotyledons, absence of primary root, absent or hypertrophied shoots, cotyledons with dark brown spots, and necrotic roots (Figure 9).

### 3.5. Morphological Aspects of C. tapia Young Plant

**Figure 10:**
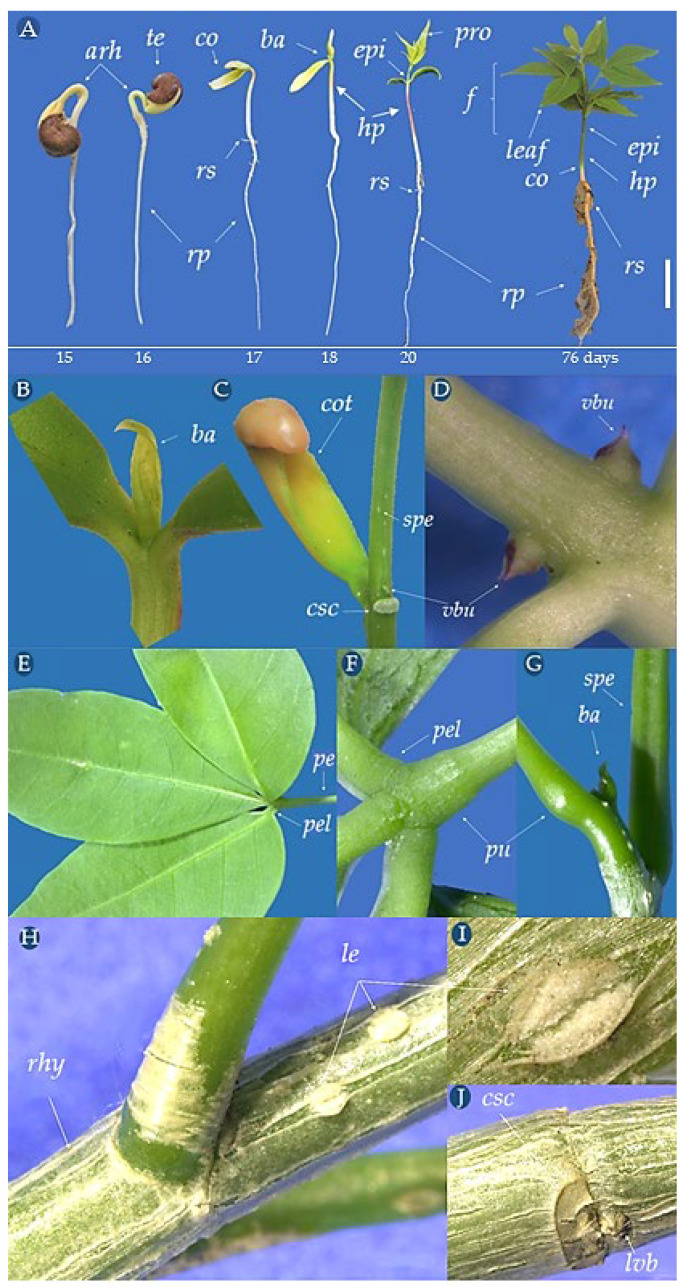
Seedling and young plant aspects of *C. tapia*. Seedling development to young plant (**A**), apical bud (**B**), cotyledons (**C**), vegetative bud (**D**), leaflets (**E**), pulvinus (**F**), petiole (**G**), rhytidome (**H**), lenticels (**I**), cotyledonary scar (**J**). *Arh*–hypocotyledonary arch, *cot*–cotyledons, *csc*–cotyledon scar, *ep*–epicotyl, *le*–leaves, *leaf*–leaflets, *ab*–apical bud, *vbu*–lateral vegetative bud, *hp*–hypocotyl, *le*–lencicelles, *ap*–absorbent, *pe*–petiole, *pel*–petiolule, *spe*–sulcate petiole, *pu*–pulvin, *pr*–primary root, *sr*–secondary root, *lvb*–lateral vegetative bud. Scale bar = 4.0 cm.

The *C. tapia* seedlings’ emergence until the young plant stage took 81 days after sowing. After 15 days of sowing, the hypocotyledon arch was above the substrate, and after 16 days, the presence of an integument adhered to the cotyledons emerging from the soil was observed. After 17 days, the cotyledons were released from the integument and began to expand. After 18 days, one of the cotyledons was erect, and the other with the end adhered to the ground exposing the plumule. After 20 days, the seedling was completely formed with roots, hypocotyl, distended cotyledons, epicotyl, and a pair of trifoliate leaves (Figure 10A).

After 76 days, the young plant had a well-developed primary root of approximately 15 cm in length. The axial root was sinuous, cylindrical, yellowish to light rust color, becoming woody with a hyaline root cap. Secondary roots were fine, sinuous, cylindrical, tender, abundant, and well distributed along the primary root; tertiary roots were less abundant. The roots showed simple, white absorbent hairs, only seen under a dissecting microscope. The collar was cylindrical and glabrous with a whitish or greenish color. The hypocotyl was 6.5 cm and the epicotyl 2.0 cm in length, with five alternate leaves, the third leaf centered, and the protophils emerging from the apical meristem.

The young stem was straight, cylindrical, some with a dark green periderm and others reddish and glabrous, with some whitish lenticels and the cotyledons abscised after 81 days, becoming yellowish, leaving a clear scar (Figure 10C). In the axillary stem region, a dormant, small, green, triangular, and almost imperceptible vegetative bud was observed (Figure 10D), which became rusty in a more advanced stage of development.

The pair of perpendicular leaflets was asymmetrical to the lobes of the limbus. The central leaflet was elliptical, wider in the middle, and narrow towards the end (Figure 10E). The marginal portion showed subtle wavy indentations, with a spitting apex and a wedged base with pulvinulus on the abaxial surface, where the petiole is short and grooved, with a slight pulvinus at the base at both ends (Figure 10G), and the apical bud was greenish with hyaline ends. *C. tapia* showed compound trifoliolate, with complete morphology leaves, formed by a sheath, petiole, and limb, with herbaceous peninerve leaflets, glabrous surface, inert, smooth, and hyaline edge.

Eighty-six days after sowing, the young plant had a total length of 25 cm, from the root cap to the primordia leaf. The stem had a 3.72 cm circumference and good leaf development, with six branches. Visible lenticels were observed in the extension of the peridermis, marked longitudinal rhytidomas (characterized by longitudinal or transversal fissures that form several plaques in the stem), and the dormant vegetative buds persisted and dried near the cotyledon scar (Figure 10J).

## 4. Discussion

The results obtained in this study demonstrate broad intraspecific phenotypic variability in the biometric and morphological characteristics of fruits, seeds, seedlings, and young plants of *C. tapia*. This variability reinforces the usefulness of the analyses performed not only for selecting fruits based on specific traits, but also for supporting germplasm conservation efforts and guiding seed collection programs. Additionally, the data contribute to a better understanding of the species’ reproductive potential, providing a basis for conservation practices, seedling production, and ecological restoration in degraded areas.

Biometric differences are commonly observed in fruits and seeds of native plants, which demonstrates the high gene flow of the species and the influence of edaphoclimatic variations [32]. A species with high variability has a high genetic potential for germplasm conservation and seed collection [33]. The afore-cited authors found variations in the size of ripe fruits of *Byrsonima verbascifolia* Rich. ex A. Juss. Different responses for fruit biometrics may be related to the fruit maturation stage and the collection site [34].

The coefficient of variation (CV) for width of seeds was considered low, and for the other variables, it was considered medium; both are within the range proposed by Pimentel-Gomes [35], which reported the coefficients of variation as low if less than 10% and medium if between 10 and 20%. Such statistical parameters are essential to compare the quantitative aspects of random distributions of biometric traits, supporting descriptive analysis and statistical inference research to compare plant populations from different environments and plant breeding studies [14].

Seed thickness was the trait with the highest coefficient of variation, as observed in other biometric studies, such as with *Poincianella rpyramidalis* (Tul.) L.P. Queiroz [36]. The width had less dispersion than length, differently from what was observed in *Capparis flexuosa* L. seeds [14]. The seeds of *C*. *tapia* are considered small, as proposed by Santos et al. [37]; when the seed mass was divided by the fruit mass, it was found that *C. tapia* seeds represent approximately 3% of the total biomass allocation, indicating that the photoassimilate flux during development was established in greater proportion in the fruit. Also, according to these authors, these differences in biomass distribution between the fruit structures result from the fruit’s structural needs due to drainage. In addition, it may also be a response of the mother plant to the diversity in the composition of photoassimilates for better fruit development. Mota et al. [38] observed high phenotypic variability in fruits and seeds of *Dipteryx alata* Vogel, particularly among mother plants within subpopulations. Differences were more pronounced for fruit traits than for seed traits, indicating potential for conservation and genetic improvement. Fruit size showed a strong correlation with seed size, suggesting its suitability as a field selection criterion. Rehling et al. [39] demonstrated that wide subindividual variation in fruit size is a distinctive feature in species bearing small fruits, enhancing connectivity with different frugivores at both individual and population levels. However, this variability may impose ecological constraints related to the size of dispersal gaps, exerting selective pressures on fruit size, especially under scenarios of large-frugivore loss. The study thus highlights how morphological variation in fruits influences plant–frugivore interactions and contributes to coevolutionary processes in small-fruited species.

In the present study, no detailed assessment of the mother plants’ habitats was conducted, which limits the direct confirmation of environmental influence on the observed variability. Nevertheless, statistical analysis of biometric traits revealed high coefficients of variation, particularly for fruit mass (CV = 28.44%) and number of seeds (CV = 36.15%), as well as considerable values for seed thickness (CV = 17.19%) and seed mass (CV = 14.10%). Moreover, distinct patterns of skewness and kurtosis indicated significant heterogeneity among sampled individuals, evidencing broad phenotypic plasticity within the subpopulations of *C. tapia*. Similar results have been reported in studies with *Eugenia dysenterica* DC., in which a wide phenotypic variability in fruits and seeds was recorded. The authors emphasized that this diversity is relevant for plant improvement and can be used as an indicator of population structuring in the species’ natural range. These findings reinforce the importance of biometric characterizations for understanding variability within native species [40].

The fruits of *C*. *tapia* are of the amphisarcid type, with a globose, obovoid or oblong shape, indehiscent, polysemy and without a replum, similar to those of other species of the same family. The species *Neocalyptrocalyx longifolium* (Mart.) Cornejo & Iltis. (Capparaceae) is also an amphissarcidium fruit, oblong, and yellow at maturity, that measures 3.8 to 7.5 by 1 to 3.5 cm [41]. The fruit and seed size variation is expected in species located in the Caatinga biome [42] and may derive from genetic, physiological, or environmental factors [32]. When the variation is low, the plants are found in an environment of primary vegetation [43]. The larger seeds are conducive to nutrient storage during development and, consequently, well-formed embryos that will produce more vigorous seedlings [19].

The seeds of *C*. *tapia* vary in color (yellow to brown), with a kidney-shaped shape, have a woody integument with irregular tubercles, a very evident ventral hilum, and are completely surrounded by an aril, as reported by Barroso et al. [26]. The reniform shape is commonly observed in *Capparaceae* and *Cleomaceae* (sister taxon) species such as *Capparidastrum frondosum* (Jacq.) Cornejo & Iltis, *Mesocapparis lineata* (Pers.) Cornejo & Iltis, and *Neocalyptrocalyx longifolium* (Mart.) Cornejo & Iltis. Their seeds are all smaller than 2.3 cm [38], and particularly for the *Tarenaya* genus, the seeds measure approximately 0.25 mm (length) by 0.15 mm (width) [44]. Inside the seed, between the apex of the radicle and the ventral cotyledon, an intercellular space is probably filled with air, which gives the integument a milky color, as explained by Barroso et al. [26].

The initial water content of *C*. *tapia* seeds after processing and a three-day natural drying period was 10.2%, a value expected for intermediate seeds, which do not tolerate desiccation below 10.0–12.5% [45]. The water absorption curve of *C*. *tapia* has a triphasic behavior for seeds incubated at temperatures of 20 to 35 °C. The triphasic pattern of seed germination is characterized by three distinct phases: rapid initial water absorption (phase I), metabolic stabilization with cellular reactivation (phase II), and, finally, new absorption associated with radicle protrusion (phase III) [46]. Seeds submitted to the temperature of 40 °C did not enter into cell division. Consequently, their meristems did not elongate and deteriorate [47].

*C*. *tapia* seeds germinated best at 30–35 °C, did not form normal seedlings at 20 °C, and showed low and late germination at 40 °C. Thus, this indicates that temperature affects germination, indicating that it interferes with the speed of biochemical reactions responsible for regulating the entire metabolic process in the seed. There is, therefore, an optimal temperature that promotes a higher germination percentage and rate, as well as maximum and minimum thresholds at which the process still occurs, albeit less efficiently [19]. Farooq et al. [48] reported that increasing temperature favored germination up to a maximum point, beyond which a gradual decline was observed. Similarly, studies such as those by Moura et al. [49], which evaluated the effect of temperature on the germination of *Commelina benghalensis* L. and *Richardia brasiliensis* Gomes seeds, observed increased germination within specific thermal ranges and reduction at extreme temperatures, confirming the sensitivity of seeds to thermal variations. Some authors reported seed germination inhibition at lower or elevated temperatures, such as 25 °C in *C. tapia*, which had 42% germination [50], null at 40 °C for *Myrciaria jaboticaba* (Vell.) Berg, *Myrciaria cauliflora* (Mart.) Berg, and *Myrciaria peruviana* var. trunciflora [51], and also null at 45 °C for *Dalbergia nigra* (Vell.) [52].

The temperature of 30 °C also promoted higher germination (52%) in *Tocoyena formosa* (Cham. & Schltdl.) K. Schum seeds, while the temperature of 15 °C inhibited germination [53]. Similarly, when the *C. tapia* seeds were submitted to extreme temperatures (20 and 40 °C), the germination was negatively affected. The germination percentage of *C. tapia* seeds was low compared to the values obtained by Galindo et al. [50], in which seeds from the same population had germination between 84 and 95%. This difference may be related to several factors, such as seed size, plant nutritional status, environmental factors, and interannual variation.

Generally, the physiological quality of seeds in forest species is related to size. Hence, in the same lot, the lowest germination and vigor values are obtained from smaller seeds, which is usually associated with the location in which the population is inserted [9,37]. Some studies corroborate this information, such as for *Macrolobium acaciifolium* (Benth.) seeds, where more vigorous seedlings were obtained from larger seeds [54]. In a complementary yet unpublished study, it was observed that seeds from a particular mother plant exhibited superior performance both under controlled conditions (Germination = 92%) and in the field (Emergence = 80%). When compared to their morphobiometric characteristics, these seeds were classified in the second group in terms of size and mass. However, this plant produced fruits with greater size, mass, and number of seeds. The germination of *C*. *tapia* is epigeal-phanerocotyledonary, starting after 96 h with the emission of the white primary root, and presents fleshy and green cotyledons, with rare cases of seedlings with three cotyledons, possibly due to mutation. The presence of several cotyledons is a common feature in *Gymnospermae* seeds but uncommon in Angiospermae. In a report by Gurgel [55] with the polyembryonic species *Syzygium jambos* L., three to four cotyledons in one of the embryos were easily counted, some with different sizes, as observed for *C. tapia*. Cotyledons have important traits that can aid in taxonomic and phylogenetic studies between plant groups, in addition to the identification of species [56].

In *C*. *tapia*, 12 days after sowing, seedlings with several anomalies were observed, including absence of structures, spots on the cotyledons, and necrotic roots. Abnormalities are characterized when the seedlings have no fundamental structure or deformation during the initial germination development [16]. Identifying abnormalities in seedlings is important because it indicates a negative effect on the physiological potential of the seed lot [57]. In addition, these seedlings generally are incapable of continuing their development and developing into normal plants under adequate conditions [23].

Seedling abnormalities occur due to seed damage, poor embryo formation [58], pathogen infection, and unfavorable environmental conditions during maturation [59]. In the plant life cycle, the post-seminal phase is critical due to its importance in field propagation [60]. When distinguishing normal and abnormal seedlings, the development pattern of the species under study must be considered [61]. In *Hyptis cana* Pohl., seedlings with dark-colored protophills, without roots or only a deteriorated portion, were considered abnormal [62].

*C*. *tapia* seedling emergence occurred gradually, with the seedling fully formed by 20 days, presenting roots, hypocotyl, epicotyl, and trifoliate leaves. Throughout development, the young plant displayed well-distributed roots with absorbent hairs, a straight, glabrous stem, and the shedding of cotyledons, leaving a noticeable scar. The senescence of cotyledons in *C. tapia* contradicts the information proposed by Smith [56], where the lifespan of fleshy cotyledons is shorter, of 2 to 3 weeks. The leaves of *C*. *tapia* are compound, trifoliate, with a sheath, petiole, and blade, presenting peninerve, herbaceous, glabrous, unarmed, smooth leaflets with a hyaline edge. Among the Brazilian *Capparaceae*, trifoliate leaves are only reported for *C. tapia* [38].

The morphological and physiological characterization of *C. tapia* fruits, seeds, and seedlings provides essential information for understanding its life cycle, germination strategies, and initial development, allowing for the definition of appropriate conditions for the propagation and conservation of the species. Such knowledge is fundamental for restoration and management programs in the Caatinga, as it favors the maintenance of plant diversity, the recovery of degraded areas, and the protection of the ecosystem as a whole, ensuring the survival not only of *C*. *tapia*, but also of the community of organisms that depend on it.

## 5. Conclusions

The present study provides a significant contribution to the morphological and germinative knowledge of *C. tapia*, a native species of the Caatinga biome with recognized ecological, medicinal, and ornamental importance. Biometric analyses of fruits and seeds revealed wide variability in size, mass, and number of seeds per fruit, reflecting the influence of both genetic and environmental factors on the species. This diversity is essential for maintaining the adaptive potential of *C. tapia* and should be considered in conservation and ecological restoration strategies.

Fruit biometrics predominantly ranged as follows: fruit length between 37.6 and 47.5 mm (68%), fruit width between 43.5 and 48.4 mm (44%), fresh fruit mass between 41.8 and 56.7 g (39%), and number of seeds per fruit between 15 and 22 (46%). Seed biometrics showed predominant values of length between 7.8 and 9.5 mm (68%), width between 7.2 and 8.0 mm (61%), thickness between 4.2 and 5.1 mm (54%), and mass between 0.13 and 0.18 g (84%).

Germination characterization showed that temperature exerts a strong influence on the process, with the range of 30–35 °C being the most favorable for the development of normal seedlings. In contrast, extreme temperatures (20 °C and 40 °C) reduced germination and seed vigor, indicating marked thermal sensitivity. This information is essential for guiding seedling production protocols in nurseries and for reforestation programs aimed at restoring degraded areas in the Brazilian semiarid region.

Observations on seedling morphology and the identified anomalies, such as embryos with three cotyledons, provide important insights for establishing a standardized classification of anomalies in native species, facilitating future studies on morphological and physiological variations. Moreover, the presence of anomalies may indicate both genetic variation and environmental effects during seed development.

This study also has direct practical applications. For conservation purposes, the information obtained can guide seed collection from different populations and at various times, ensuring greater genetic variability in ecological restoration programs. In nurseries, it is recommended to use seeds collected from mature and healthy fruits, with germination conducted at 30 °C under controlled humidity and light conditions to obtain vigorous and uniform seedlings.

In agriculture and sustainable use, understanding the reproductive cycle and optimal germination conditions of *C. tapia* promotes its propagation for ornamental, medicinal, and forage purposes. Knowledge of seed size and vigor allows for the selection of lots with higher physiological potential, optimizing seedling production. Furthermore, the observed biometric variability may support genetic improvement and domestication programs for native species.

Thus, this study provides detailed and unprecedented morphological descriptions of *C. tapia*, offering a foundation for biodiversity conservation and the sustainable use of plant resources in the Caatinga biome, contributing to ecological restoration and the valorization of native Brazilian species.

## Figures and Tables

**Table 1:** Descriptive statistics of fruit length (Length), fruit width (Width), fruit fresh mass (Mass), and the number of seeds per fruit (NumS) in *Crateva tapia*.

Statistical Parameters	Length (cm)	Width (cm)	Mass (g)	NumS
Mean	4.33	4.37	51.47	19.12
Standard deviation	0.46	0.43	14.64	6.91
Maximum	5.33	5.42	97.59	40.00
Minimum	3.25	3.34	26.69	7.00
Skewness ^(1)^	−0.11	−0.11	0.63	0.72
Kurtosis +3 ^(2)^	2.54	2.66	3.30	3.11
Shapiro–Wilk ^(3)^	0.63 ^ns^	0.62 ^ns^	0.01 *	0.00 **
CV (%)	10.71	9.83	28.44	36.15

^(1), (2)^ Asymmetry and Kurtosis differ from 0 and 3, respectively; ^(3)^ *^,^ ** Significant up to 1 and 5% probability, respectively. ^ns^ Not significant.

**Table 2:** Descriptive statistics of seed length (Length), seed width (Width), seed thickness (Thick), and fresh mass (Mass) of *Crateva tapia* seeds.

Statistical Parameters	Length (cm)	Width (cm)	Thick (cm)	Mass (g)
Mean	0.85	0.74	0.49	0.15
Standard deviation	0.09	0.69	0.08	0.02
Maximum	1.08	0.90	0.78	0.23
Minimum	0.68	0.53	0.32	0.09
Skewness ^(1)^	0.40	−0.47	0.93	0.23
Kurtosis +3 ^(2)^	2.77	3.62	4.76	3.96
Shapiro–Wilk ^(3)^	0.08 ^ns^	0.02 *	0.00 **	0.31 ^ns^
CV (%)	10.20	9.38	17.19	14.10

^(1), (2)^ Asymmetry and kurtosis differ from 0 and 3, respectively. ^(3)^ *, ** Significant up to 1 and 5% probability, respectively. ^ns^: Not significant.

**Table 3:** Analysis of variance summary for germination percentage (G), germination first count (GFC), germination speed index (GSI), root length (RL) and shoot (SL), root dry mass (RDM) and shoot (SDM), hard seeds (HardS), and abnormal seedlings (AbS) of *Crateva tapia* submitted to different temperatures.

SV	DF	Mean Squares								
		G (%)	GFC (%)	GSI	RL (cm)	SL (cm)	RDM (g)	SDM (g)	HS (%)	AbS (%)
Temp.	2	508.0833 **	306.583 **	0.44066 **	0.00352 **	0.00905 **	0.00001 **	0.00004 **	14.33333 ^ns^	1.75000 ^ns^
Residue	9	6.61111	3.75000	0.00579	0.00037	0.00092	5.30555	0.00001	7.88889	3.19444
Total	11	–	–	–	–	–	–	–	–	–
CV (%)	–	9.95	19.53	9.13	18.31	11.61	9.18	14.60	23.09	54.99

** significant up to 1% probability; ^ns^ not significant; SV = source of variation; DF = degree of freedom; Temp. = temperatures; CV (%) = coefficient of variation.

**Table 4:** Germination (G), germination first count (GFC), germination speed index (GSI), root (RL) and shoot length (SL), root (RDM), shoot dry mass (SDM), hard seeds (HS), and abnormal seedlings (AbS) of *Crateva tapia* under different temperatures.

Temp. (°C)	G (%)	GFC (%)	GSI	RL (cm)	SL (cm)	RDM (g)	SDM (g)	HS (%)	AbS (%)
25	30 c	1 c	0.49 c	0.011 b	0.022 b	0.001 b	0.004 b	14 a	4 a
30	50 a	10 b	1.15 a	0.013 a	0.031 a	0.004 a	0.010 a	10 a	3 a
35	34 b	19 a	0.85 b	0.007 c	0.025 b	0.001 b	0.005 b	13 a	4 a
CV (%)	9.95	19.53	9.13	18.31	11.61	9.18	14.60	23.09	54.99

Means followed by the same letter in the columns do not differ by the Tukey test at 5% probability.

## Data Availability

The original contributions presented in the study are included in the article. Further inquiries can be directed to the corresponding authors.
